# Smart Poly(imidazoyl-l-lysine): Synthesis and Reversible Helix-to-Coil Transition at Neutral pH

**DOI:** 10.3390/polym9070276

**Published:** 2017-07-11

**Authors:** Estefania Piedra-Arroni, Fatma Makni, Laura Severac, Jean-Luc Stigliani, Geneviève Pratviel, Colin Bonduelle

**Affiliations:** 1 CNRS, LCC (Laboratoire de Chimie de Coordination (UPR8241)), 205 route de Narbonne, F-31077 Toulouse 31400, France; estefania.piedra@gmail.com (E.P.-A.); maknifatma27@gmail.com (F.M.); laura.severac@gmail.com (L.S.); stigliani@lcc-toulouse.fr (J.-L.S.); Genevieve.Pratviel@lcc-toulouse.fr (G.P.); 2 Université Paul Sabatier, Université de Toulouse, 118 Route de Narbonne, Toulouse 31062, France

**Keywords:** smart polypeptides, imidazole-containing polymers, helix-to-coil transition, poly(lysine), pH-responsive

## Abstract

Polypeptide polymers can adopt natural protein secondary structures such as α-helices or β-sheets, and this unique feature is at the origin of some intriguing physico–chemical properties. In this work, we present how side chain imidazoylation of a poly(l-lysine) scaffold affords the preparation of poly(histidine) counterparts exhibiting α-helix conformation. This structuring behavior is reversible and can be controlled by means of pH and or temperature changes.

## 1. Introduction

Synthetic polypeptide polymers are made of natural building blocks (amino acids), and they bring important breakthroughs in materials sciences applications including those requiring smart polymers, i.e., polymers able to respond to external stimuli [1,2,3]. Smart polypeptides are ideal candidates to mimic adaptive biological systems such as natural proteins, by undergoing structural or conformational changes in response to biologically relevant external stimuli including other molecules or the environment (temperature, pH, redox…) [4]. Indeed, synthetic polypeptide polymers can reproduce natural protein secondary structures including α-helices or β-sheet structures [5]. Compared to polymers presenting a coil structure, structured polypeptides exhibit intriguing physico–chemical properties either in bulk, at the surface, or in solution [6]. Compared to natural proteins, polypeptidic polymers can easily undergo secondary structure transitions that can be easily implemented and tuned by tailoring amino acid side chains [5]. For instance, helix to coil transitions can be controlled by means of pH changes for poly(l-glutamic acid) PGA [7,8] and poly (l-lysine) PL [9]. Recent developments in this direction include the use of polypeptide polymers to enable a structuring switch upon biologically relevant stimuli changes [1,10,11,12,13], including redox changes [14,15], metal coordination [16] or DNA binding [17].

Imidazole-containing polymers are an important class of smart materials that possesses remarkable properties [18,19]. They are extensively used to develop responsive nanocarriers, which are utilized in biomedical applications such as anti-cancer drug delivery (response to tumor extracellular pH) and nucleic acid delivery (response to endosomal pH) [20]. Among imidazole-containing polymers, poly(histidine) (PHIS) is a well-known macromolecule made of histidine amino-acids [20,21]. PHIS displays imidazole moieties on its lateral chains, which can be protonated at a pKa value between 6 and 7. This pKa value is unique within natural amino acids, and the amphoteric nature of PHIS in physiological conditions has led to materials exhibiting sol–gel responsiveness, endosome disruption properties, or theranostic applications [18]. So far, synthetic methodologies that are involved in PHIS synthesis have involved protected *N*-carboxyanhydride (NCA) monomers that require drastic synthetic schemes, and which have often led to significant racemization upon deprotection [22]. Aside from the use of NCA derivatives of histidine, imidazole-containing polypeptides can also be prepared by post-polymerization coupling [23], and previous works have introduced imidazole moieties onto the lateral chains of polypeptide polymers [23,24]. Generally, one big challenge of the post-polymerization approach relies on the achievement of full grafting density, which is favored for structured polypeptide polymers [25]. To the best of our knowledge, previous attempts have permitted the introduction of imidazole moieties with high but incomplete grafting density, and no study has revealed the resulting structure of the polymer after imidazole grafting [24]. 

In this work, we have prepared a small library of poly(imidazoyl-l-lysine) (PIL) grafted with imidazole moieties spanning 0% to 99% grafting density. The structure of the different PILs was studied in detail by circular dichroism and by infrared spectroscopy, and our results showed that PILs are homopolymers that can be structured as an α-helix if high grafting density is achieved. This is in marked contrast to poly(l-histidine) blocks, which have been shown recently to adopt a highly aggregating β-sheet structure when lateral chains are deprotonated [22]. Herein, we also show that this α-helix structure is reversible by means of pH changes and temperature changes. Overall the structure difference between PHIS and PIL (1) may have a crucial impact in biology in a context where the α-helix may present unique biological properties [26]; and (2) should now be taken into account if both materials are compared for drug or gene delivery [18].

## 2. Materials and Methods

Carboxylic acid of 4-imidazoleacetic acid (IAA), *N*,*N*′-dicyclohexylcarbodiimide (DCC), *N*-hydroxysuccinimide (NHS), triethylamine (TEA), methanol, dimethylsulfoxide (DMSO), acetone, diethyl ether, sulfuric acid (H_2_SO_4_) and sodium hydroxide (NaOH) were purchased from Sigma-Aldrich (Darmstadt, Germany) and used without further purification. *N*-carboxyanhydride monomer (NCA) of trifluoroacetyl-l-lysine was purchased from Isochem (Vert-le-Petit, France). Propargylamine (98%) was purchased from Sigma-Aldrich and double distilled before use. *N,N*-Dimethylformamide (DMF) was obtained from a Solvent Purification System (SPS) and freshly used for the polymerization. Nuclear Magnetic Resonance (NMR) spectra were recorded on a Bruker Avance spectrometer (Bruker, Germany). Chemical shifts are reported relative to the deuterated solvents used (CDCl_3_, D_2_O). For structuring studies, diluted solutions were prepared with Milli-Q water. The pH of every sample was measured with a Mettler Toledo SevenCompact^TM^ S220 pHmeter (Mettler Toledo, France), calibrated with Mettler Toledo buffer solutions between pH = 4 and pH = 10. The circular dichroism (CD) measurements were performed on a JASCO J-815 spectropolarimeter (JASCO, Oklahoma City, OK, USA) between 195 and 260 nm (far-UV), by using a quartz cell of 1 cm path length, at the desired temperature (20 °C for standard measurements, or a temperature gradient between 10 and 80 °C for special measurements). The measure parameters were optimized as follows: sensitivity between 5 and 200 mdeg, 0.01 mdeg resolution, 8 s response time (Digital Integration Time), 1 nm bandwidth and 10 nm/min scanning rate. The polypeptide solutions were diluted with Milli-Q water. The pH of the solutions was adjusted either by using H_2_SO_4_ or NaOH aqueous solutions (0.1 M). Detailed descriptions of the synthetic procedures, circular dichroism analyses and molecular dynamic simulations can be found within the supporting info part (ESI). 

## 3. Results and Discussion

Considering previous works dealing with imidazole post-polymerization coupling onto polypeptide, poly(l-lysine) (PL) was chosen, as it previously afforded high grafting density [23,24]. First, the polypeptide scaffold was obtained in two steps. 

**Scheme 1:**
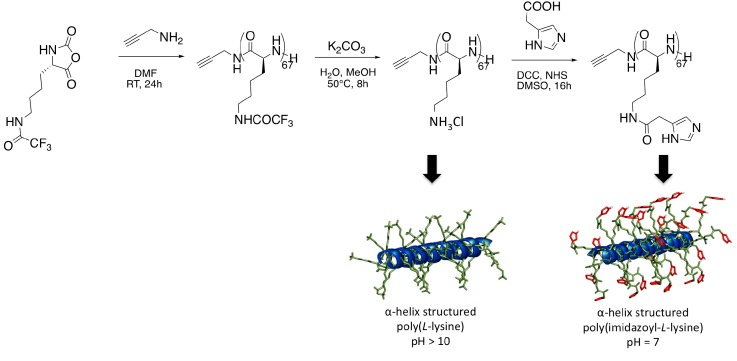
Poly(imidazoyl-l-lysine) synthesis: Towards helix structured imidazole derivatives of poly(l-lysine).

The first step involved the controlled ring-opening polymerization of *N*-ε-trifluoroacetyl-*l*-lysine-*N*-carboxyanhydride, initiated by propargylamine in DMF at room temperature (see Scheme 1). Size exclusion chromatography analysis in DMF evidenced a low polydispersity index of 1.14 for a *M_n_* value of 18,600 g·mol^−1^ (see Figure S1a in ESI). In a second step, poly(*N*-ε-trifuoroacetyl-l-lysine) lateral chains were deprotected using smooth basic conditions to avoid racemization of the backbone. Extensive dialysis afforded a poly(l-lysine) polymer that was characterized by both NMR and size exclusion chromatography (SEC) performed in water (see ESI and Figure S1b). A polymerization degree of 67 was determined by ^1^H NMR analysis by comparing integrals of the proton signal of the initiator (propargylamine, 3.9 ppm) and the signal of the polypeptide backbone (COCHNH, 4.3 ppm, see Figure S1b in ESI) and SEC performed in aqueous conditions provided a *M_n_* value of 21,500 g·mol^−1^ and a low polydispersity index of 1.15. Then, aqueous solutions of PL were prepared at pH values of between 4 and 8 (150 μM in monomer units). Circular dichroism of the solutions further revealed an extended structure (coil structure) for all these pH values, as the spectra presented a positive and maximum Δε value at λ = 216 nm (see ESI Figure S5).

**Figure 1:**
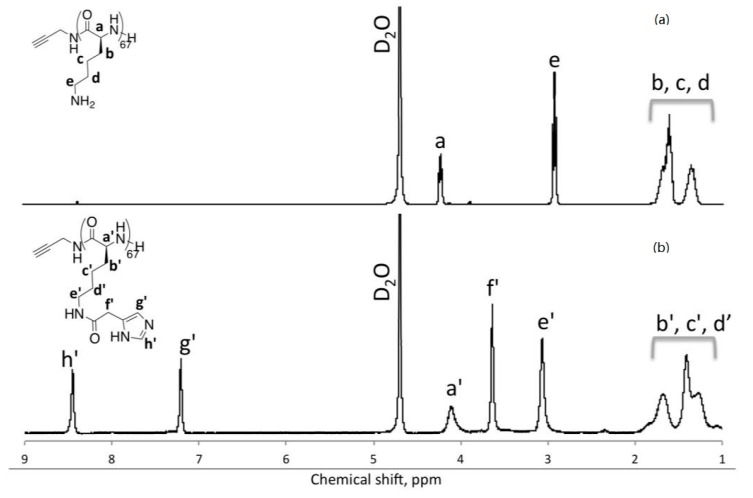
^1^H NMR spectra in D_2_O: (**a**) poly(l-lysine); (**b**) poly(imidazoyl-l-lysine).

A small library of 4 poly(imidazoyl-l-lysine) polymers with various grafting densities of imidazole was then obtained by using post-polymerization coupling with 4-imidazoleacetic acid (IAA). The peptidic coupling involved free amine lateral chains of the poly(l-lysine) (Scheme S1) and the carboxylic acid moiety of IAA. The coupling was performed in anhydrous dimethylsulfoxyde conditions by using in excess, and concomitantly, *N,N*′-dicyclohexylcarbodiimide (DCC) and *N*-hydroxysuccinimide (NHS) as coupling agents (Scheme S2). After purification, NMR and SEC analysis were used to evaluate imidazole grafting by (1) calculating the grafting density from NMR analysis (integration of the signal a’ from the polypeptide backbone at 4.1 ppm, as compared to the signal g’ of the imidazole ring at 7.2 ppm; see Figure 1 and Table 1; and (2) checking the change in elution time after coupling by size-exclusion chromatography (ESI, Figure S2). Overall, 4 different poly(imidazoyl-l-lysines) were prepared as PIL **1**–**4**, with 31%, 47%, 75% and 99% imidazole grafting respectively (Table 1 and Figure S4 in ESI).

**Figure 2:**
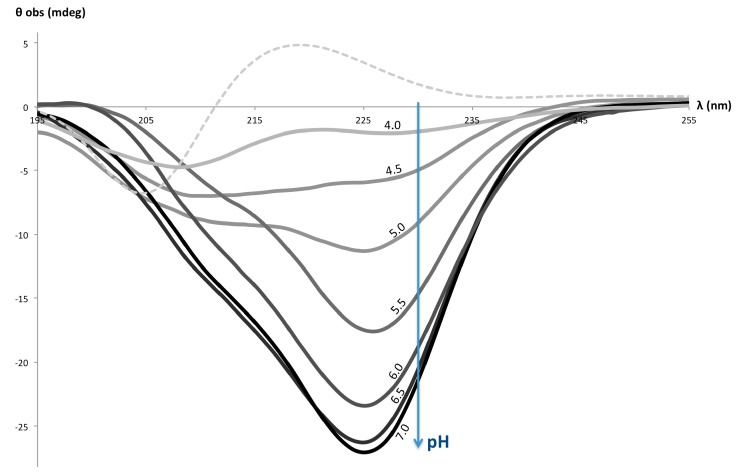
CD spectra at 150 μM in aqueous solutions of poly(l-lysine) at pH = 7 (dashed line) and of poly(imidazoyl-l-lysine) at pH values comprised between 4 and 7 (other traces).

We further screened the structure of PIL **1**, which was fully functionalized with imidazole moieties. Different polymer solutions were prepared in water (150 μM in monomer units), and the pH was carefully adjusted. As depicted in Figure 2, CD spectra were recorded between 195 and 255 nm at different pH values between 4 and 7. In marked contrast to the spectra of PL (dashed line at pH 7), the spectra of PIL revealed significant changes in CD with the appearance of minimum values that were attributable to an alpha helix structure [16]. Overall, the CD signature of the helix displayed a significant smaller 208 nm minimum and a slight red shifted n–π * peak at 225 nm (as compared to 222 nm) that previous work have attributed to the occurrence of alpha helix nanoaggregation [27,28]. It should be mentioned here that the minimum values obtained above pH = 6 correspond to a [θ] value at 222 nm of −17.6 × 10^−3^ mdeg·cm^2^·dmol^−1^. Moreover, FTIR spectra of PIL **1** strengthen the structuring behavior observed by CD: IR spectra displayed amide bands typical of α-helical conformations (1654 and 1545 cm^−1^, see Figure S3 ESI).

**Figure 3:**
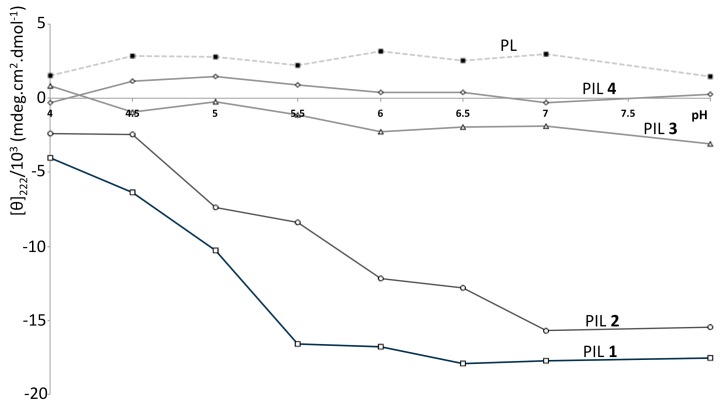
pH influence on structuring of PIL **1**–**4** (pH between 4 and 8, molar ellipticities calculated from θ_obs_ at 222 nm).

We then studied helix formation with PIL **2** to **4** by monitoring CD spectra in aqueous solutions at the same concentration and at different pH values between 4 and 8. As clearly depicted in the Figure 3, the influence of the grafting density was significant, and only PIL **1** and **2** exhibited structuring above a pH of 6. As the [θ] values became more important with increasing amounts of imidazole units, the grafting density was assigned to the structuring of the polypeptide block. Overall, CD analyses pointed out that a significantly high grafting density had to be achieved to form secondary conformations at a pH comprised between 6 and 8.

**Figure 4:**
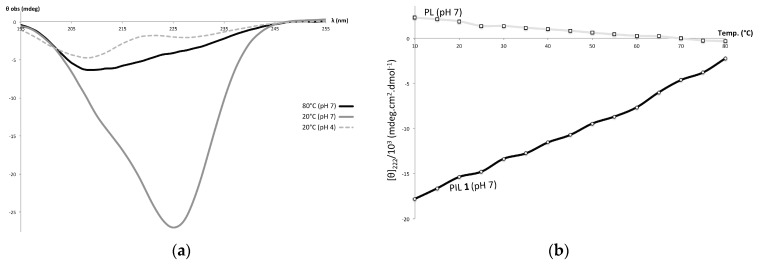
(**a**) CD spectra of poly(imidazoyl-l-lysine) at 20 °C versus 80 °C (pH of 7 and 150 uM); (**b**) Temperature as a destructuring trigger of PIL **1** (pH 7, molar ellipticities calculated from θ_obs_ at 222 nm).

Pursuing the design of smart polypeptide systems, we then focused on helix destructuring by means of temperature changes (Figure S6 in ESI). In fact, and as previously reported with PL alone, an increase in temperature triggers helix-to-β sheet transition, a rare feature with polypeptide polymers. Therefore, CD spectra of PIL **1** in an aqueous solution (pH = 7) were recorded at a temperature range from 10 to 80 °C. Molar ellipticities taken from these analyses, at 222 nm, were plotted, as shown in Figure 4. Significant helix destructuring was indeed observed when the temperature was increased. Nevertheless, at 80 °C, the CD spectra did not reveal structuring in beta sheet conformation, meaning that the post-polymerization coupling inhibits this PL structuring behavior.

Previous studies on multicharged polypeptides, such as PGA and PL, have established that their reversible helix-to-coil transition was attributable to the electric repulsion of their respective side chains [8,9]. When charged, the repulsion of lateral chains breaks the hydrogen network at the origin of the helix structure. This structure is then recovered when the side chain charges are controlled, for instance, by pH changes. This phenomenon is certainly at the origin of the helix-to-coil transition observed with poly(imidazoyl-l-lysine) backbones. To strengthen this point, molecular modelling was performed with GROMACS in order to evaluate how the hydrogen-bonding network behaves with or without charges at the imidazole lateral chain extremities. Upon dynamic calculations (see ESI Figures S7 and S8), the presence of positive charges at the imidazole ring significantly disturb the hydrogen-bonding network of the polymer, as compared to the same hydrogen-bonding network without charges at the lateral chains extremities. This theoretical result prompted us to conclude that electronic repulsion is also at the origin of the structuring changes observed for poly(imidazoyl-l-lysine). Current studies in our laboratory include PIL helix structuring to design a smart metallopolypeptide [16] in a context where its poly(l-histidine) counterpart affords the production of polypeptide adopting beta sheet conformations [22].

## 4. Conclusions

As simplified analogues of natural proteins, synthetic polypeptide polymers constitute important tools for developing new promising applications, particularly if smart systems can be designed. In this paper, we showed that, thanks to imidazole group introduction onto the lateral chains of a poly(l-lysine), the helix-to-coil transition of the polypeptide occurs around a neutral pH value. This result appears to be important for two reasons: (1) poly(l-histidine) polymers adopt beta sheet conformations and poly(imidazoyl-l-lysine) is therefore a helical counterpart of these polymers; (2) imidazole-containing polypeptides hold tremendous promise in drug and gene delivery, and it is noteworthy that the structuring behavior presented in this work occurs in the pH range of their promising bioactive properties. 

## Figures and Tables

**Table 1:** Influence of the IAA stoichiometry to control imidazole grafting density.

Polymer	IAA Used for Coupling ^a^	% Imidazole Grafting ^b^	Yield of the Coupling Step	Mn ^d^ (PDI)
PL	-	0	-	21,500 (1.15)
PIL 1	2 eq.	>99 ^c^	85%	38,400 (1.17)
PIL 2	1 eq.	75	84%	nd ^e^
PIL 3	0.6 eq.	47	86%	nd ^e^
PIL 4	0.3 eq.	31	89%	nd ^e^

^a^ As compared to monomer units stoichiometry, eq. means equivalent; ^b^ calculated from ^1^H NMR spectrum using the signals e and e’ (cf Figure 1 and ESI); ^c^ no residual e signal observed; ^d^ in g/mol; ^e^ nd: not done.

## Supplementary Materials

Supplementary materials (ESI) are available online at www.mdpi.com/2073-4360/9/7/276/s1.
