# Supplementary material for: Smart Poly(imidazoyl-l-lysine): Synthesis and Reversible Helix-to-Coil Transition at Neutral pH
Source: Polymers (Basel). 2017 Jul 11;9(7):276. doi: 10.3390/polym9070276 (PMC6432093; doi:10.3390/polym9070276)
Supplement: Supplementary file 1 [file polymers-09-00276-s001.pdf]

Communication

Smart poly(imidazoyl-*L*-lysine) : reversible helix-to-coil transition at neutral pH

Estefania Piedra-Arroni, Fatma Makni, Laura Severac, Jean-Luc Stigliani, Geneviève Pratviel, Colin Bonduelle*

Dr. E. Piedra-Arroni, F. Makni, L. Severac, Dr. J.L. Stigliani, Dr. G. Pratviel, Dr. C. Bonduelle; CNRS, LCC (Laboratoire de Chimie de Coordination (UPR8241)
205 route de Narbonne, F-31077 Toulouse, France.
E-mail: colin.bonduelle@lcc-toulouse.fr.

---

1. Materials and Methods

Carboxylic acid of 4-imidazoleacetic acid (IAA), N,N'-dicyclohexylcarbodiimide (DCC), *N*-hydroxysuccinimide (NHS), triethylamine (TEA), methanol, dimethylsulfoxide (DMSO), acetone, diethyl ether, sulfuric acid (H_2SO_4) and sodium hydroxide (NaOH) were purchased from Sigma-Aldrich and used without further purification. *N*-carboxyanhydride monomer (NCA) of trifluoroacetyl-*L*-lysine was purchased from Isochem. Propargylamine (98%) was purchased from Sigma-Aldrich and double distilled before use. DMF was obtained from a Solvent Purification System (SPS) and freshly used for the polymerization. NMR spectra were recorded on Bruker Avance spectrometer. Chemical shifts are reported relative to the deuterated solvents used ($CDCl_3$, D_2O). Diluted solutions were prepared with Milli-Q water. The pH of every sample were measured with a Mettler Toledo SevenCompact^{TM} S220 pHmeter, calibrated with Mettler Toledo buffer solutions between pH = 4 and pH = 10. The circular dichroism measurements were performed on a JASCO J-815 spectropolarimeter between 195 nm and 260 nm (far-UV), by using a quartz cell of 1 cm path length, at the desired temperature (20 °C for standard measurements, or a temperature gradient between

10 °C and 80 °C for special measurements). The measure parameters were optimized as follows: sensitivity between 5 and 200 mdeg, 0.01 mdeg resolution, 8 seconds response time (Digital Integration Time), 1 nm bandwidth and 10 nm/ min scanning rate. The polypeptide solutions were diluted with Milli-Q water. The pH of the solutions was adjusted either by using H_2SO_4 or NaOH aqueous solutions (0.1 M).

2. Synthetic procedures

1.2.1. <u>Poly(*L*-lysine) synthesis :</u>

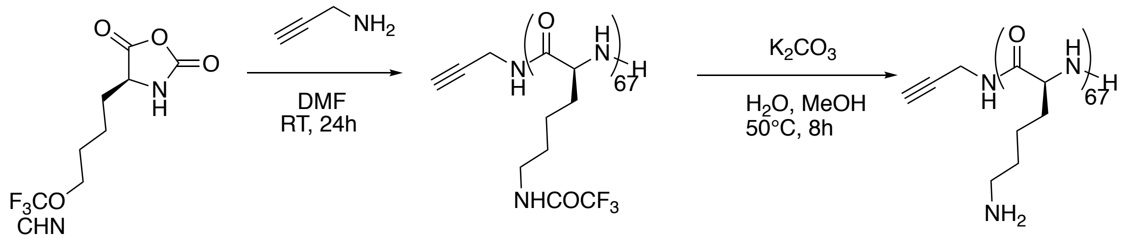

Scheme S1. Poly(L-lysine) synthesis in two steps

Poly(trifluoroacetyl-L-lysine) preparation. In this work, propargylamine was used to initiate the ring-opening polymerization of *N*-Trifluoroacetyl-*L*-lysine *N*-carboxyanhydride (see scheme S1). This initiation step would permit to produce a clickable poly(trifluoroacetyl-*L*-lysine) block that can be further used for chemical ligation.[1] The NCA monomer of trifluoroacetyl-*L*-lysine (2 g, 7.5 mmol) was weighed in a glovebox under argon, introduced in a flamedried schlenk, and dissolved with 5 mL of anhydrous DMF. The solution was stirred for 10 min, and propargylamine (8 µL, 0.12 mmol) was added with an argon purged syringe. The solution was stirred for 24h at room temperature under argon. The polymer was then recovered by precipitation in diethylether and dried under high vacuum. Yield: 95%. Molar masses were determined by SEC in DMF/LiBr (1%) using an Acquity Advanced 4 Polymer Chromatography System (Waters) equipped with an Acquity APC XT column for extended temperature organic-based separations (4.6 Å~ 150 mm) and an Acquity Refractive

Index detector. Calibration was performed by using polystyrene standards. The sample was dissolved in DMF and was run at a flow rate of 0.5 mL.min^{-1} at 55 °C.

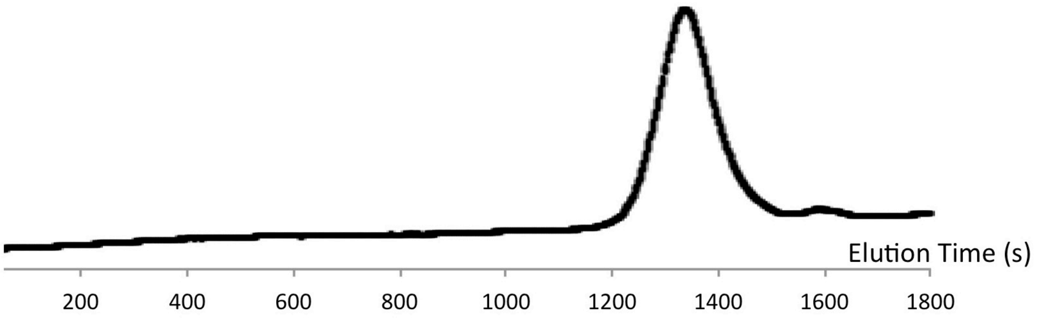

Figure S1a. SEC trace of the poly(trifluoroacetyl-*L*-lysine) (elution in DMF with 1% LiBr, detection by RI)

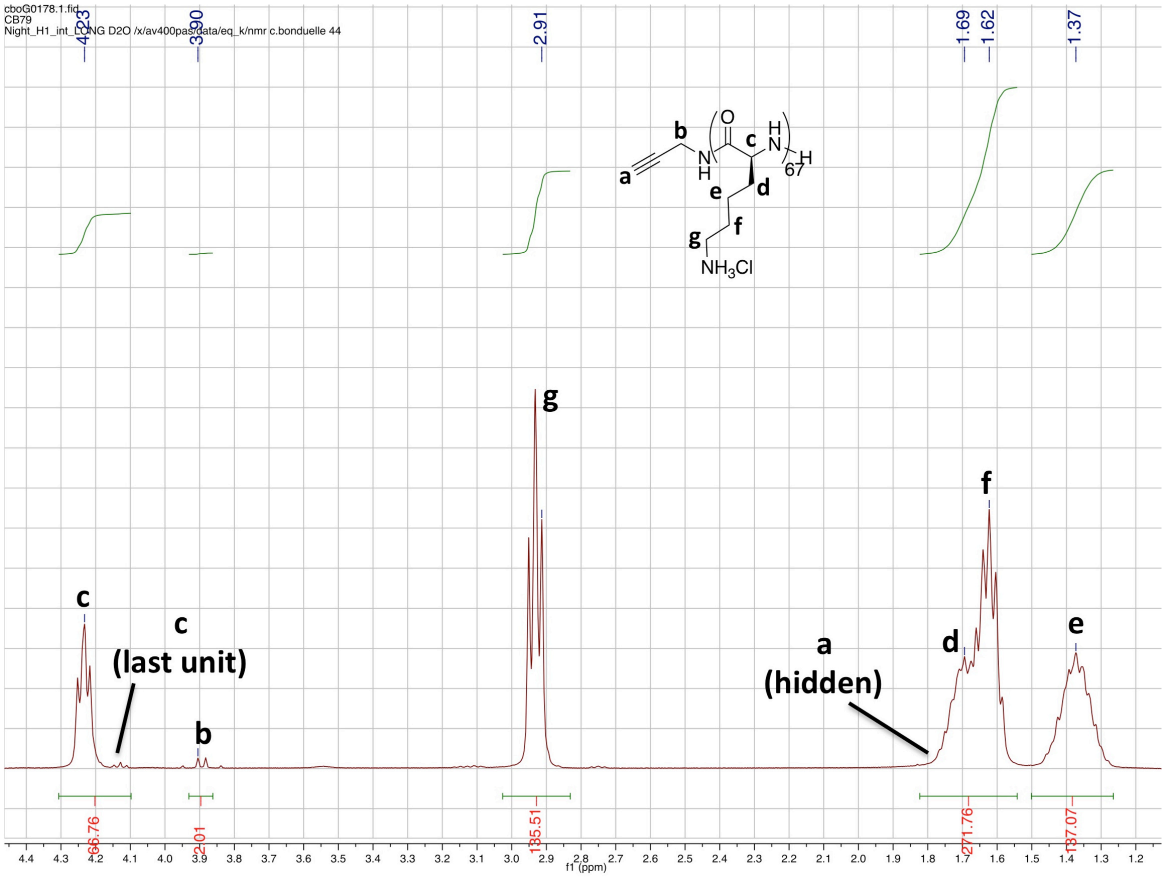

Figure S1b. ^{1}H NMR spectra of poly(L-lysine) in D_2O upon dialysis.

Poly(trifluoroacetyl-L-lysine) deprotection. A round-bottom flask was charged with 400 mg of the protected polypeptide in 30 mL of a 1:9 (v/v) mixture of H_2O and MeOH. The suspension was heated to 50°C, and K_2CO_3 (580 mg, 2 eq./lateral chains) was added to the reaction mixture. During the course of the reaction, the initial suspension turns into a homogeneous solution. After 8 h, the reaction mixture was cooled to room temperature and solvents were removed under vacuum. The residue was suspended in water (8 mL) and a saturated NaCl solution (9.5 mL). The resulting solution was dialyzed against milliQwater (MWCO 2 kDa) and lyophilized to give chloride salt of poly(*L*-Lysine) as a white solid. Yield: 93%. FT-IR (main bands, cm^{-1}): ν = 3331 (w; NH), 1656 (s; C=O in amide group), 1536 (s; NH in amide group); ^{1}H NMR of the polypeptide backbone (400 MHz, D_2O+DCl (2%), δ, ppm): 4.23 (m, 67H, COC**H**NH), 3.90 (d, 2H, CCC**H₂**NH), 2.91 (m, 135H, C**H₂**NH₃Cl), 1.80-1.55 (m, 271H, CHC**H₂**CH₂ and C**H₂**CH₂NH₃Cl) 1.23-1.42 (m, 137H, 2H, CH₂C**H₂**CH₂). ^{13}C NMR of the polypeptide backbone (100.6 MHz, D_2O+DCl (2%), δ, ppm): 173.5 (**C**ONH), 53.3 (CO**C**HNH), 39.1 (**C**H₂NH₃Cl), 30.5 (CH**C**H₂CH₂), 26.2 (**C**H₂CH₂NH₃Cl), 22.1 (CH₂**C**H₂CH₂).

1.2.2. Poly(imidazoyl-*L*-lysine) synthesis :

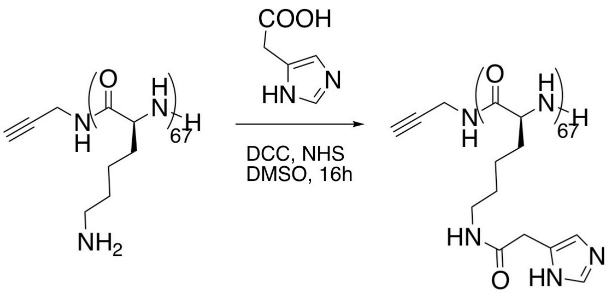

Scheme S2. Post-polymerization coupling with IAA

It is to note that our procedure was a slight optimization of a procedure already published[2] : this previous published procedure involved the use of HOBt 1) that turned to be

not efficient in our case and 2) whose signals were incorrectly assigned by Haam and coworkers to the imidazole ring.[2] In our work, imidazole-grafted poly-*L*-lysine (PIL) were synthesized by conjugation of the ε-amine of PL and the carboxylic acid of 4-imidazoleacetic acid using concomitantly N,N'-dicyclohexylcarbodiimide (DCC) and N-hydroxysuccinimide (NHS) as coupling agents.

*Synthesis of PIL **1** and general procedure for the preparation of PIL **2-4***: the desired amount of 4-imidazoleacetic acid (63 mg, 0,39 mmol, 2 equiv. per ε-amine of PL) was dissolved in 3 mL of DMSO. Molar excess amounts of DCC (241 mg, 1.17 mmol, almost 3 equivalent per carboxylic acid units) and then NHS (157 mg, 1.37 mmol, almost 3.5 equivalent per carboxylic acid units) were added to the suspension. After few minutes, reaction mixture became yellow then orange. After 1 hour of stearring, triethylamine (50.6 µL, 0.36 mmol) was added and a white precipitate appeared. PL (25 mg, 0,19 mmol of ε-NH2) was added and the mixture was stirred for another 16 h at room temperature. Resulting suspension became brown and was centrifugated (20 min., 3000 rpm). Surnatant was then precipitated in a mixture of acetone/diethyl ether (3/1). The resulting precipitate was solubilized in water and dialyzed against milliQwater (MWCO 2 kDa) before to be lyophilized to give poly(imidazoyl-*L*-Lysine) as a brown solid. Yield: 85%. FT-IR (main bands, cm^{-1}, see figure SX): ν = 3331 (w; NH), 1651 (s; C=O in amide group), 1545 (s; NH in amide group); ^{1}H NMR of the polypeptide backbone (400 MHz, D_2O+DCl (2%), δ, ppm): 8.45 (s br, 68H, Im), 7.20 (s br, 64H, Im) 4.13 (m, 67H, COC**H**NH), 3.63 (m, 131H, ImC$\mathbf{H_2}$CO), 3.06 (m, 135H, C$\mathbf{H_2}$NHCO), 1.78-1.56 (m, 141H, C$\mathbf{H_2}$$CH_2$NHCO) 1.56-1.14 (m, 271H, CHC$\mathbf{H_2}$$CH_2$ and CH_2C$\mathbf{H_2}$$CH_2$). ^{13}C NMR of the polypeptide backbone (100.6 MHz, D_2O+DCl (2%), δ, ppm): 173.6 (**C**ONH), 169.5 (**C**ONH lateral chains), 133.1 (Im), 126.8 (Im), 117.6 (Im), 53.7 (CO**C**HNH), 39.4 (**C**H_2NHCO), 31.5 (CO**C**H_2Im), 27.4 (CH**C**$H_2$$CH_2$), 25.6 (**C**$H_2$$CH_2$$NH_3$CO), 21.8 ($CH_2$**C**$H_2$$CH_2$). Grafting density was determined by ^{1}H NMR

analysis by comparing integrals of the proton signals at 7.20 ppm (coming from IAA) and the proton signal at 4.13 ppm (coming from the backbone). Molar masses were also determined by SEC in aqueous solutions at 50 °C using an Acquity Advanced 4 Polymer Chromatography System (Waters) equipped with with three Styragel columns (Ultrahydrogel 120, Ultrahydrogel 250, and Ultrahydrogel 1000). The eluent was a pH 3.3 buffer solution comprising 0.3 M of NaH_2PO_4 and 1.0 M of acetic acid at a flow rate of 0.6 mL min^{-1}. Standard PEG with precise molecular weights from SigmaeAldrich were used for column calibration.

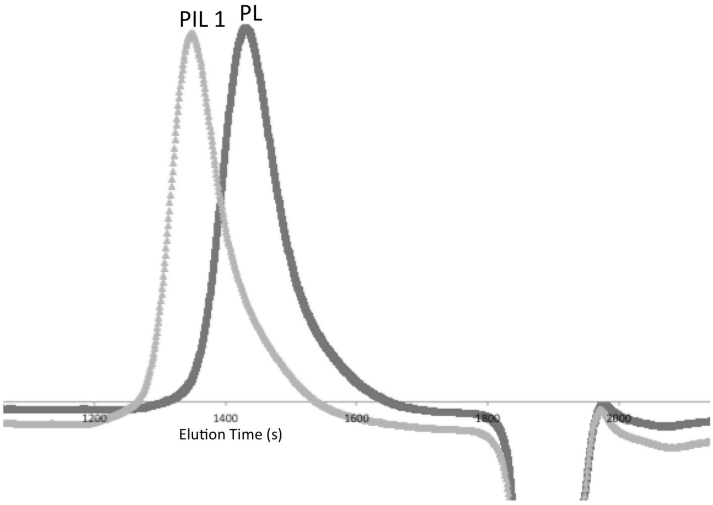

Figure S2. SEC trace of the poly(*L*-lysine) PL and the fully grafted poly(imidazoyl-*L*-lysine) PIL **1** (elution in pH 3.3 buffer solution comprising 0.3 M of NaH_2PO_4 and 1.0 M of acetic acid and detection by RI).

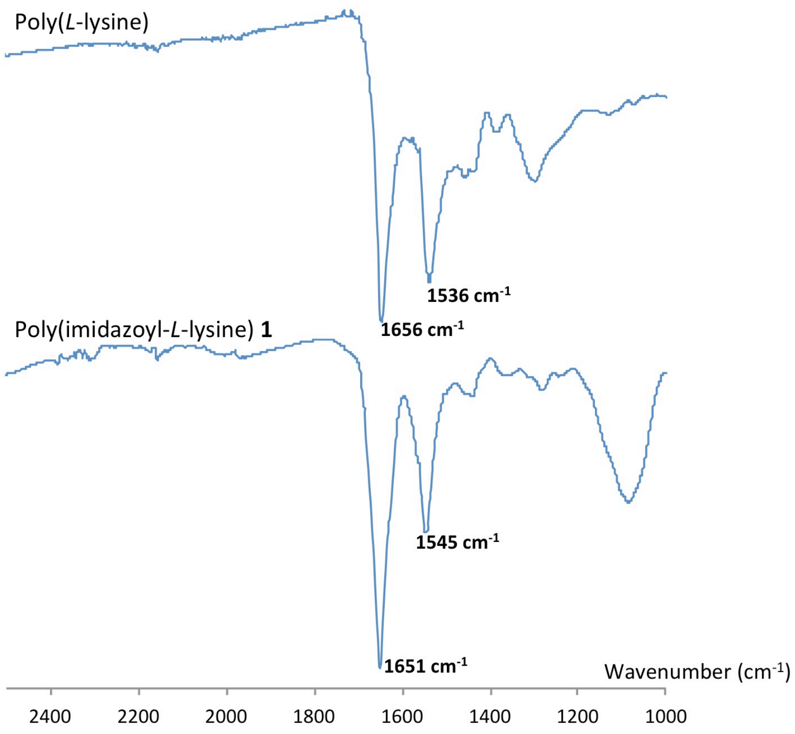

Figure S3. FTIR of PIL **1** (top) as compared to FTIR of PL before imidazoylation (down)

*Synthesis of PIL **2*** : This polypeptide was prepared by the same method as described above for PIL **1** except that only 1 equivalent of IAA relative to the lateral chains of PL was used. DCC and NHS were indeed adjusted accordingly. Yield : 84 %. NMR of the polypeptide backbone (400 MHz, D_2O+DCl (2%), δ, ppm): 8.45 (s br, 50H, Im), 7.20 (s br, 51H, Im) 4.13 (m, 67H, COC**H**NH), 3.63 (m, 101H, ImC**H$_2$**CO), 3.06 (m, 109H, C**H$_2$**NHCO), 2.88 (m, 33H, C**H$_2$**NH_3Cl), 1.75-1.53 (m, 171H, C**H$_2$**CH_2NHCO) 1.49-1.14 (m, 241H, CHC**H$_2$**CH_2 and CH_2C**H$_2$**CH_2). ^{13}C NMR of the polypeptide backbone (100.6 MHz, D_2O+DCl (2%), δ, ppm): 173.6 (**C**ONH), 169.5 (**C**ONH lateral chains), 133.1 (Im), 126.8 (Im), 117.6 (Im), 53.7 (CO**C**HNH), 53.3 (CO**C**HNH PL), 39.4 (**C**H_2NHCO), 39.1 (**C**H_2NH_3Cl PL), 31.5 (CO**C**H_2Im), 30.5 (CH**C**H_2CH_2 PL), 27.4 (CH**C**H_2CH_2), 26.2 (**C**$H_2CH_2NH_3$Cl PL), 25.6 (**C**$H_2CH_2NH_3$CO), 22.1 (CH_2**C**H_2CH_2 PL), 21.8 (CH_2**C**H_2CH_2). Grafting density was determined by ^{1}H NMR analysis by comparing integrals of the proton signals at 3.63 ppm

(coming from IAA) and the proton signal at 4.13 ppm (coming from the backbone). *Synthesis of PIL **3*** : This polypeptide was prepared by the same method as described above for PIL **1** except that only 0.6 equivalent of IAA relative to the lateral chains of PL was used. DCC and NHS were indeed adjusted accordingly. Yield : 86 %. NMR of the polypeptide backbone (400 MHz, D_2O+DCl (2%), δ, ppm): 8.45 (s br, 29H, Im), 7.20 (s br, 30H, Im) 4.13 (m, 67H, COC**H**NH), 3.63 (m, 64H, ImC**H₂**CO), 3.06 (m, 62H, C**H₂**NHCO), 2.88 (m, 74H, C**H₂**NH₃Cl), 1.77-1.53 (m, 206H, C**H₂**CH₂NHCO) 1.49-1.19 (m, 193H, CHC**H₂**CH₂ and CH₂C**H₂**CH₂). ^{13}C NMR of the polypeptide backbone (100.6 MHz, D_2O+DCl (2%), δ, ppm): 173.6 (**C**ONH), 169.5 (**C**ONH lateral chains), 133.1 (Im), 126.8 (Im), 117.6 (Im), 53.7 (CO**C**HNH), 53.3 (CO**C**HNH PL), 39.4 (**C**H₂NHCO), 39.1 (**C**H₂NH₃Cl PL), 31.5 (CO**C**H₂Im), 30.5 (CH**C**H₂CH₂ PL), 27.4 (CH**C**H₂CH₂), 26.2 (**C**H₂CH₂NH₃Cl PL), 25.6 (**C**H₂CH₂NH₃CO), 22.1 (CH₂**C**H₂CH₂ PL), 21.8 (CH₂**C**H₂CH₂). Grafting density was determined by ^{1}H NMR analysis by comparing integrals of the proton signals at 3.63 ppm (coming from IAA) and the proton signal at 4.13 ppm (coming from the backbone). *Synthesis of PIL **4*** : This polypeptide was prepared by the same method as described above for PIL **1** except that only 0.3 equivalent of IAA relative to the lateral chains of PL was used. DCC and NHS were indeed adjusted accordingly. Yield : 89 %. NMR of the polypeptide backbone (400 MHz, D_2O+DCl (2%), δ, ppm): 8.45 (s br, 22H, Im), 7.20 (s br, 23H, Im) 4.13 (m, 67H, COC**H**NH), 3.63 (m, 40H, ImC**H₂**CO), 3.06 (m, 45H, C**H₂**NHCO), 2.88 (m, 91H, C**H₂**NH₃Cl), 1.79-1.54 (m, 230H, C**H₂**CH₂NHCO) 1.49-1.18 (m, 191H, CHC**H₂**CH₂ and CH₂C**H₂**CH₂). ^{13}C NMR of the polypeptide backbone (100.6 MHz, D_2O+DCl (2%), δ, ppm): 173.6 (**C**ONH), 169.5 (**C**ONH lateral chains), 133.1 (Im), 126.8 (Im), 117.6 (Im), 53.7 (CO**C**HNH), 53.3 (CO**C**HNH PL), 39.4 (**C**H₂NHCO), 39.1 (**C**H₂NH₃Cl PL), 31.5 (CO**C**H₂Im), 30.5 (CH**C**H₂CH₂ PL), 27.4 (CH**C**H₂CH₂), 26.2 (**C**H₂CH₂NH₃Cl PL), 25.6 (**C**H₂CH₂NH₃CO), 22.1 (CH₂**C**H₂CH₂ PL), 21.8 (CH₂**C**H₂CH₂). Grafting density was

determined by ^{1}H NMR analysis by comparing integrals of the proton signals at 3.63 ppm (coming from IAA) and the proton signal at 4.13 ppm (coming from the backbone).

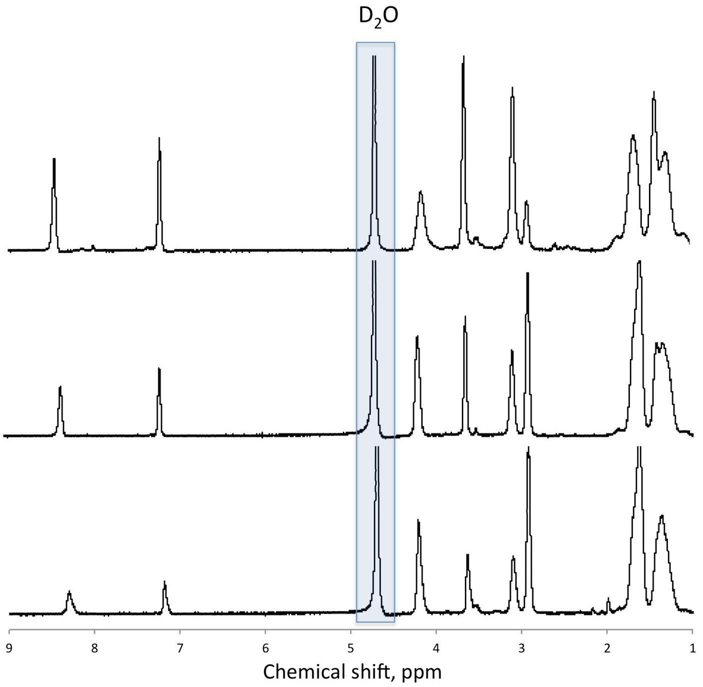

Figure S4. ^{1}H NMR spectra of poly(imidazoyl-*L*-lysine) **2** (top), **3** (middle), **4** (down)

3. Circular Dichroism Spectroscopy

1.3.1. <u>Generalities:</u>

The secondary structure of proteins/peptides (α-helix, β-sheet…) can be analyzed from the CD spectra in the range from 195 to 255 nm, corresponding to the peptide bond absorption. The secondary structure of the polymers was studied by CD spectroscopy using the following procedure: the final concentration (the concentration in the cuvette used for the CD analyses) was always 150 μM in monomer units. The final volume in the cuvette was always 1 mL (pathlength of 10 mm). The pH was verified and eventually adjusted once samples prepared and just before CD analysis. According to previous report,[3] PL structured in

α-helix present two minima both at 208 and 222 nm. The “molar ellipticity” also called the “mean residue ellipticity” has been calculated as follow:

$[Q] = (10 \times Q_{obs}) / (l \times c)$

[Q] is expressed in $deg.cm^2.dmol^{-1}$ and Q_{obs} was the observed ellipticity in degrees (deg), l is the path length in dm, and c is the polypeptide concentration in mol/L.

The Q_{obs} at 222 nm wavelenght was chosen as it is a recognized minimum for alpha helix structuring.[4] It is to note that specific work done previously with other polypeptides have showed that this minimum may strongly depends on the molecular weight and on the solubility.[5]

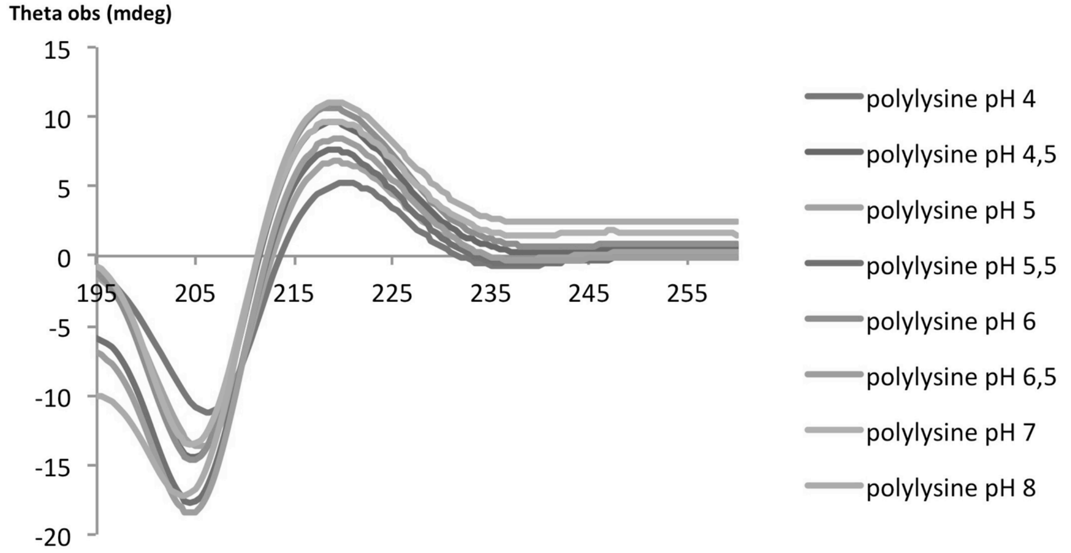

Figure S5. CD spectra of poly(*L*-lysine) at 150 uM and at pH comprised between 4 and 8

1.3.2. <u>Preparation of samples and solutions for CD measurements :</u>

Preparation of mother solutions used for the CD experiments: PIL mother solution: the polymer solution was always prepared the day before CD analysis. The mother solution of the polymer was prepared by dissolving the desired quantity of the polymer in Milli-Q water (polymer concentration: 6 mM in monomer units). For each experience, an aliquot of the desired volume was taken off from this solution and diluted in order to keep the final concentration at 150 μM in monomer units. Typical CD solution : 25 μL of polymer mother solution (6 mM) was added in a tube, followed by 950 μL of Milli-Q water eventually mixed with few uL of aqueous solutions of NaOH 0.1 M and or H_2SO_4 0.1 M if necessary. The volume was completed to 1 mL final volume with Milli-Q water. The samples were always prepared the day before analyses. Typical CD solution for T gradients measurements by CD: 25 μL of polymer mother solution (6 mM) was added in a tube and 950 μL of Milli-Q water eventually mixed with few uL of aqueous solutions of NaOH 0.1 M and or H_2SO_4 0.1 M if necessary. The volume was completed to 1 mL final volume with Milli-Q water. The samples were always prepared the day before analyses. The temperature gradient was analyzed over a range from 10 °C to 80 °C with a scan speed of 10 °C / min. Before starting the measurements at each temperature; the sample was maintained at the desired temperature for 10 minutes in order to be sure that the analysis temperature inside the sample was reached.

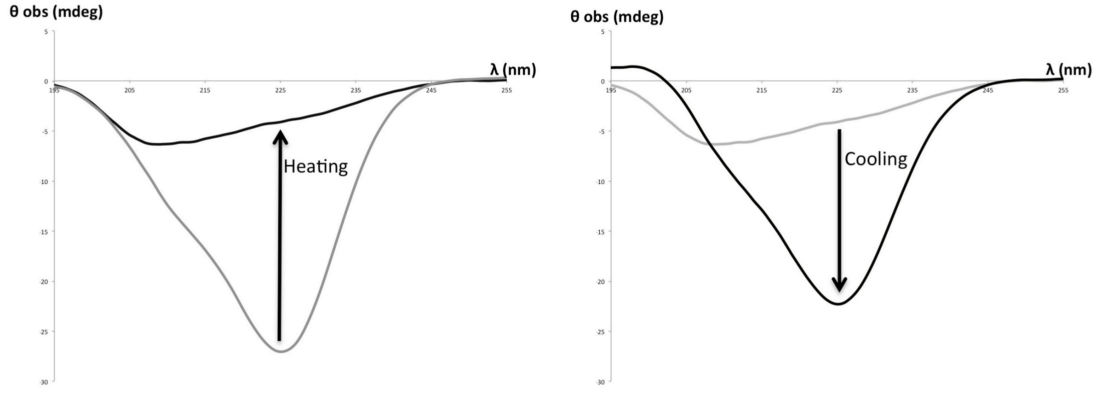

Figure S6a. CD spectra at 150 μM in aqueous solutions of poly(imidazoyl-L-lysine) at a pH value of 7 from 20°C to 80°C (left) or from 80°C to 20°C (right).

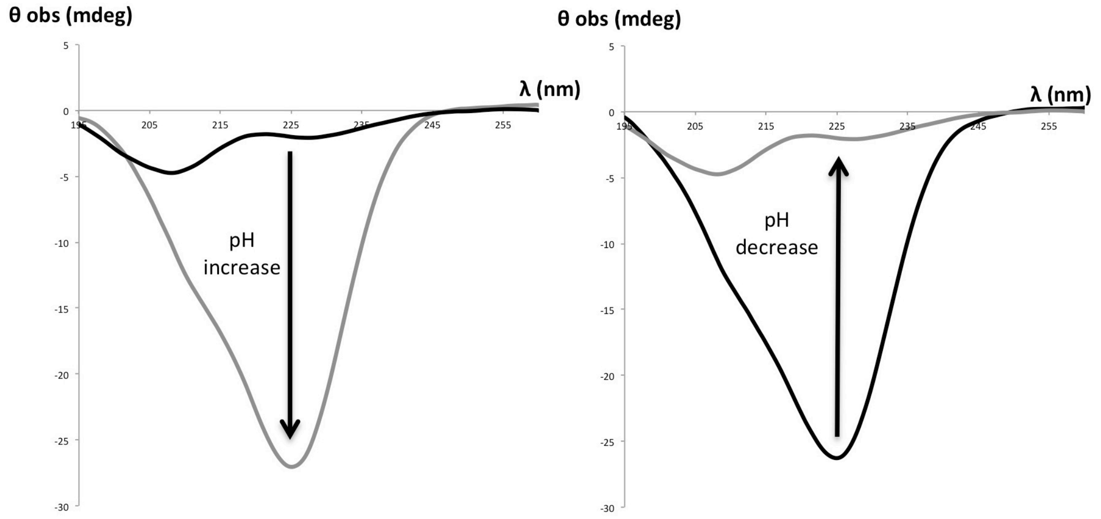

Figure S6b. CD spectra at 150 μM in aqueous solutions of poly(imidazoyl-L-lysine) from a pH value of 4 to a pH value of 7 (left) or from a pH value of 7 to a pH value of 4 (right).

4. Molecular Dynamic Simulations

The neutral and protonated monomeric imidazoyl-*L*-Lysine residues were built with the molecular modeling package Maestro 10.6.[6] The geometries were optimized using the PM7 method[7] implemented in the MOPAC 2016 quantum chemistry package.[8] Restrained electrostatic potential (RESP) charge derivation of the the two monomers was obtained from the R.E.D. server[9] and their topology was obtained from the Antechamber and Leap modules of the AmberTools16 suite.[10] The resulting library files were then used to prepare the a-helix 28-mers with Leap. N- and C-termini were acetylated and amidated, respectively, to mimic the continuation of the protein chains. The Amber topology and parameter files generated with Leap were then converted in readable GROMAC format, using the Acpype utilitie.[11] Simulations were performed with GROMACS, version 5.1.2,[12,13] using the Amber force field ff99SB-ILDN.[14] The polypeptides were solvated with SPC216 water molecules (~ 25167 water molecules) in a dodecahedron box, using periodic boundary conditions, with at least 1.1 nm between the polypeptides and the simulation box edges. In the case of the protonated 28-mer, the system was neutralized by adding 28 Cl^- ions.The two systems (neutral and

protonated) were first submitted to energy minimization with a combination of steepest descent and conjugate gradient. The equilibration stage involved 300 ps of a NVT ensemble. Solvent and solute were separately coupled to temperature baths at 300 K, with the modified Berendsen coupling[15] and a time constant of 0.1 ps. The second stage of equilibration involved 300 ps of a NPT ensemble. The Parrinello-Rahman approach[16] was used to keep the pressure at 1 bar with a relaxation time of 2.0 ps and an isothermal compressibility of 4.5 × 10^{-5} bar^{-1}. During the equilibration stage, heavy atoms of the solute were harmonically restrained with restraint forces of 1000 $kJ.mol^{-1}.nm^{-2}$.Equilibration stage was followed by an unconstrained simulation of both systems for 100 ns. The simulations were done using a time step for integration of 2 fs. The Particle-Mesh Ewald (PME)[17] long range electrostatics was applied using 16 Å for the maximum gridspacing for the Fast Fourier Transform and a cutoff distance of 10 Å for both Lennard-Jones and Coulomb potentials. H-bonds were constrained using the LINCS algorithm.[18]

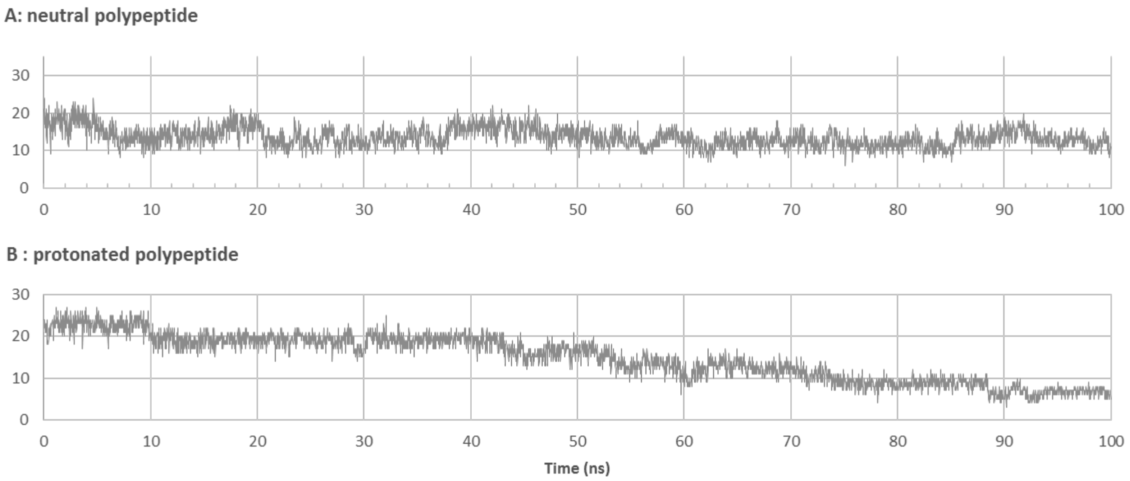

FIGURE S7: Number of hydrogen bonds versus time recorded in the peptide backbones for the neutral (A) and protonated (B) poly(imidazoyl-*L*-Lysine).

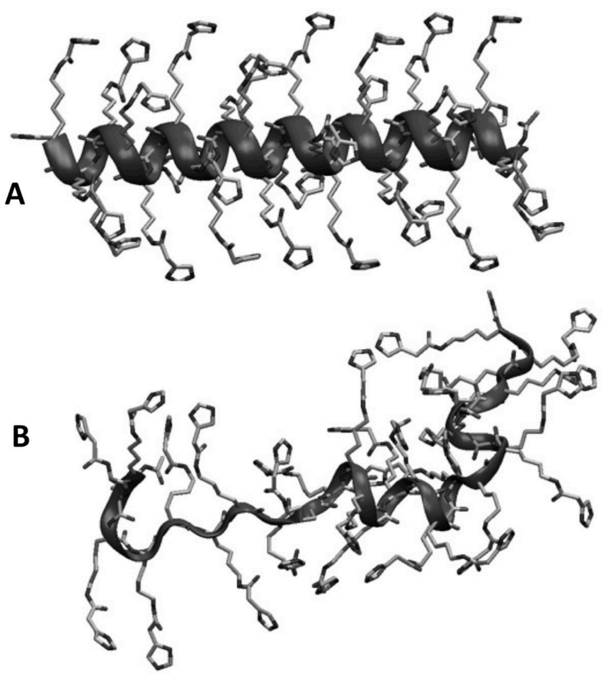

FIGURE S8: evolution of the polypeptide geometries during the course of the molecular dynamics. A) geometrie of the helices of neutral and protonated models at the beginning of the dynamics. B) geometrie of the protonated model at 100 ns. In this case, the alpha helix progressively unfolded throughout the dynamics ; only two turn of helix remained at the end of the trajectory.

REFERENCES

1. C. Schatz, S. Louguet, J.F. Le Meins, S. Lecommandoux, *Angewandte Chemie International Edition*, **2009**, 48, 2572.

2. S. Haam and coworkers, *Biomaterials*, **2013**, 4327.

3. T.P. Johnston and coworkers, *JPP*, **2002**, 54, 315.

4. G.D Fasman, "*Poly-alpha amino acids*", Decker, New York, **1967**.

5. M. Rinaudo and A. Domard, *J. Am. Chem. Soc.*, **1976**, 98, 6360.

6. Schrödinger Release 2016-4: Maestro, Schrödinger, LLC, New York, NY, **2016**.

7. J.J.P Stewart, *J. Mol. Model.*, **2013**, 19, 1.

8. J.J.P. Stewart, Computational Chemistry, Colorado Springs, CO, USA. http://OpenMOPACnet.

9. E. Vanquelef, S. Simon, G. Marquant, E. Garcia, G. Klimerak, J. C. Delepine, P. Cieplak and F.-Y. Dupradeau, *Nucl. Acids Res.*, **2011**, 39, W511-W517.

10. D.A. Case, R.M. Betz, D.S. Cerutti, T.E. Cheatham, III, T.A. Darden, R.E. Duke, T.J. Giese, H. Gohlke, A.W. Goetz, N. Homeyer, S. Izadi, P. Janowski, J. Kaus, A. Kovalenko, T.S. Lee, S. LeGrand, P. Li, C. Lin, T. Luchko, R. Luo, B. Madej, D. Mermelstein, K.M. Merz, G. Monard, H. Nguyen, H.T. Nguyen, I. Omelyan, A. Onufriev, D.R. Roe, A. Roitberg, C. Sagui, C.L. Simmerling, W.M. Botello-Smith, J. Swails, R.C. Walker, J. Wang, R.M. Wolf, X. Wu, L. Xiao and P.A. Kollman (**2016**), AMBER 2016, University of California, San Francisco.

11. A. W. Sousa de Silva, and W. F. vranken, *BMC Research Notes*, **2012**, 5, 367.

12. D. van der Spoel, E. Lindahl, B. Hess, G. Groenhof, A.E. Mark, H.J.C. Berendsen, *J. Comput. Chem.*, **2005**, 26, 1701.

13. B. Hess, C. Kutzner, D. Van Der Spoel, E. Lindahl, *J. Chem. Theory Comput.*, **2008**, 4, 435.

14. K. Lindorff-Larsen, S. Piana, K.Palmo, P. Maragakis, J.L. Klepeis, R.O. Dror, D.E. Shaw, *Proteins*, **2010**, 78, 1950.

15. G. Bussi, D. Donadio, and M. Parrinello, *J. Chem. Phys,*, **2007**, 126, 014101.

16. M. Parrinello, A. Rahman, *J. Appl. Phys.*, **1981**, 52, 7182.

17. T. Darden, D. York, L. Pedersen, *J. Chem. Phys.*, **1993**, 98, 10089.

18. B. Hess, *J. Chem. Theory Comput.*, **2008**, 4, 116.
